# Metabolic syndrome and the likelihood of knee pain and functional disability: evidence from a large middle eastern population-based study

**DOI:** 10.1186/s12891-023-06685-3

**Published:** 2023-08-04

**Authors:** Talal Ibrahim, Abdulaziz F Ahmed, Mariam Nofal, Abdelsalam Hegazy, Hassan M. K. Ghomrawi

**Affiliations:** 1 Department of Surgery, Division of Orthopaedic Surgery, Sidra Medicine, Doha, Qatar; 2 Department of Orthopaedic Surgery, Massachussetts General Hospital, Harvard Medical School, Boston, MA USA; 3 Hamad Medical Corporation, Doha, Qatar; 4 Departments of Surgery, Medicine (Rheumatology), and Pediatrics, Northwestern University Feinberg School of Medicine, 633 N St Clair, 20th Floor, Chicago, IL 60611 USA

**Keywords:** Metabolic, Syndrome, Knee, Pain, Dysfunction

## Abstract

**Objectives:**

Metabolic Syndrome (MetS) has been associated with knee osteoarthritis (KOA) in animal studies, but epidemiologic evidence of the association remains controversial. We investigated the association between MetS and knee pain and functional disability, the hallmarks of KOA, in a Middle Eastern population with high reported MetS rates.

**Methods:**

A population-based study of adult individuals was conducted between 01/2016 and 03/2019. Data collected included age, sex, blood pressure, body mass index (BMI), waist circumference (WC), and comprehensive metabolic panel blood tests. Knee symptoms were assessed using The Western Ontario and McMaster Arthritis index (WOMAC) The Adult Treatment Panel III criteria was applied to determine if participants had MetS. Multivariable regression was used to determine the association of MetS, and its components, with the WOMAC total and subscale scores.

**Results:**

Of 6,000 participants enrolled, 15.5% had MetS. The multivariate regression demonstrated that participants with MetS had significantly higher WOMAC total and subscale scores after adjusting for demographic variables; however, these associations were not significant after adjusting for BMI. Multivariate regression examining the association between MetS components and the WOMAC scores showed sex-based significant differences with WOMAC scores; however, the differences were not larger than the minimally clinical important differences.

**Conclusions:**

This study demonstrated that after adjustment for BMI, neither MetS nor its individual parameters were associated with worse knee symptoms. As such, the association between MetS and worse knee symptoms requires further study.

## Introduction

Knee symptoms such as pain, stiffness and worsening in function impose significant disability, impact quality of life, and results in significant economic burden on the health care system [1–4]. Knee pain affects up to 16.5% of men and 27.4% in women [4], and is considered a hallmark of knee osteoarthritis (KOA), the most common type of osteoarthritis.

The pathogenesis of KOA is classically attributed to degenerative processes and the wear and tear of the weight-bearing joint [2–7]. However, growing recent evidence from animal studies suggests that joint damage may also result from metabolic abnormalities (i.e., obesity, elevated serum glucose, elevated blood pressure, elevated triglyceride levels and low high-density lipoprotein cholesterol levels) known together as metabolic syndrome (MetS) [8–10]. Causal evidence between KOA and MetS in human studies remains controversial. While some studies found an association [11–14], others did not [15–17]. A recent systematic review of studies suggests that the association between MetS and osteoarthritis may be region-specific [18]. Epidemiologic evidence on this relationship from the Middle East region remains very limited.

Qatar is a Middle East country that has one of the highest rates of obesity in the world, attributed to the unhealthy diet and lifestyle [19, 20]. Estimates are that 70% of the population are overweight with a BMI ≥ 25 kg/m^2^, while 41.1% are obese with a BMI of ≥ 30 kg/m^2^ [21]. A recent survey revealed that the Qatari population has a prevalence of MetS of 26.2% [22], suggesting this population may have a high prevalence of KOA and KOA symptoms, but no population-level estimates of KOA currently exist. A cross-sectional study of 841 women aged 40–60 in Qatar found that 71.6% of women have recurring joint pain and stiffness for two weeks, of whom 50% reported having intense pain which may suggest increased prevalence of OA [23].

To fill the gap in knowledge about the relationship between MetS and knee symptoms in the Middle East region, we conducted a cross-sectional population-based study of the Qatari population to determine the association between MetS (and its components) and reporting knee symptoms. We hypothesized that there would be a significant association between MetS and increased symptom severity, and that this association would be sustained even after adjusting for BMI.

## Materials and methods

This study was reported using the STregnthening the Reporting of OBservational studies in Epidemiology (STROBE) checklist for cross-sectional studies [24].

### Study design, setting, and participants

A cross-sectional study was conducted to answer the study questions. After receiving IRB approved at our institution (study number: IRGC-01-SI-027), we surveyed participants 18 years and older who were recruited by the Qatar Biobank regarding their knee symptoms from January 2016 to March 2019. The Qatar Biobank is a population-based initiative aimed at creating a repository of biological samples and information on health and lifestyle of Qatari citizens and long-term residents. A stratified random sample of the Qatari population are invited, and their biometrics are collected through questionnaires, laboratory tests, and physical exams, which are conducted by the Biobank staff. Relevant to this study, the data included age, sex, blood pressure (BP) measurements, anthropometric measurements, comprehensive metabolic panel blood tests. For the purposes of this study, the Western Ontario McMaster Arthritis index (WOMAC) questionnaire was added to the surveys that were completed by participants.

### Data sources and variables

The main exposure (i.e., independent) variable was having MetS. We defined MetS using the widely recognized ATP III criteria [25], which determines that a person has MetS if three or more of the following are present: increased waist circumference (≥ 102 cm for males and ≥ 88 cm for females), elevated triglycerides (TG) with a value of 150 mg/dL or more, reduced high density lipo-protein (HDL) less than 40 mg/dL for males and less than 50 mg/dL for females, elevated blood pressure (BP) defined as > 130 mmHg for systolic BP and/or > 80 mmHg for diastolic BP, or elevated fasting glucose more than 100 mg/dL. These data were derived from the biometric data collected on participants.

The main outcome (i.e., dependent) variable of this study was knee symptoms as measured by the WOMAC overall score and knee pain, stiffness, and function subscale scores. The WOMAC index is a self-administered questionnaire that is validated for the evaluation of KOA [26]. It consists of 24 items that assess knee pain (5 items), knee stiffness (2 items), and knee physical function (17 items). Each item is scored on a 0 to 4 scale. The knee pain score range is 0–20, the knee stiffness score range is 0–8 and the physical function score range is 0–68. A total WOMAC score is also calculated by adding the scores of the three subscales and normalizing to 0-100 score (0 representing no knee disability, 100 representing maximum knee disability). We also collected patient demographic data, and their body mass index (BMI).

### Analysis plan

Descriptive statistics were conducted to report the patients’ characteristics. Continuous variables were reported as means and standard deviations, whereas binary categorical variables were expressed as proportions. To evaluate the relationship between those who had MetS and those who did not, bivariate analyses (T-test for continuous variables, and Chi Square test for categorical variables) were first conducted to compare the demographic characteristics and WOMAC scores of the two groups in an unadjusted fashion. Linear regression analyses were then conducted to evaluate the association between MetS and the WOMAC total, pain, stiffness, and physical scores. Two models were estimated. The first model was only age- and sex-adjusted. The second model additionally adjusted for BMI. The second model was estimated to address concerns about collinearity between BMI and MetS [27, 28], although in our patient population there was little evidence of collinearity between BMI and MetS (Pearson’s correlation coefficient = 0.32).

Linear regression models were also estimated to assess the association of MetS components with WOMAC scores. Age- and sex-adjusted linear regression models were conducted with the MetS syndrome individual components (systolic and diastolic BP, fasting blood glucose levels, HDL, and TG) as the primary predictors and WOMAC scores as the outcomes. In the regression models, each MetS individual component was included as a dichotomous variable, and a variable was considered present if it met the aforementioned ATPIII criteria for that variable.

We further explored the association between MetS and its components with WOMAC scores for each sex separately, due to well-documented metabolic differences between males and females [27, 28]. First, we performed bivariate analyses to compare the demographics and WOMAC scores between males and females. We then conducted age- and BMI-adjusted linear regression analyses to evaluate the association between the presence of MetS individual components and WOMAC scores for each sex separately. The dependent variables were the WOMAC scores, whereas the presence of MetS individual components was the primary predictor. All analyses were performed in Stata/IC (StataCorp. 2019. Stata Statistical Software: Release 16. College Station, TX: StataCorp LLC.).

## Results

A total of 6,000 participants were enrolled during the study period. All participants had complete data to determine participants’ MetS status, and to calculate their WOMAC total and subscale scores. The mean age was 39.4 +/- 13 years and 56.7% (n = 3,404) were female. The mean BMI for participants was 29.5 +/- 6.3 kg/m^2^. When the ATP III criteria were applied to the data, the prevalence of MetS was 15.5%. Participants with MetS were generally older when compared to those without MetS. In addition, the anthropometric and metabolic parameters were both significantly different between both groups, which reflected the metabolic derangement that are manifested by MetS.

### The association between MetS and WOMAC scores

Unadjusted bivariate analyses showed that participants with MetS had significantly higher WOMAC total, pain, stiffness and physical function scores when compared to those without MetS (P < 0.001) (Table 1). Age- and sex- adjusted linear regression models demonstrated that the presence of MetS was significantly associated with higher (i.e., worse) mean WOMAC total by 3.34 points, higher mean WOMAC pain by 0.69 points, higher mean WOMAC stiffness by 0.21 points, and higher mean WOMAC physical function by 2.44 points. However, this association became statistically nonsignificant when BMI was adjusted for in the regression models (Table 2).

### The association of MetS components with WOMAC scores

Linear regression models that were adjusted for age, sex and BMI demonstrated significant association between WOMAC scores and both reduced HDL and elevated FBG. The presence of elevated FBG was significantly associated with elevated WOMAC total and function scores by 1.3 and 1 points, respectively. However, elevated FBG did not significantly affect the WOMAC pain and stiffness scores. The presence of reduced HDL significantly predicted reduced (i.e. better) WOMAC total, pain, stiffness and function scores by 2.4, 0.5, 0.2 and 1.7 points, respectively; however, the presence of high TG or high BP did not significantly affect any of the WOMAC scores (Table 3).

### The association of MetS and its components with WOMAC scores while stratifying by sex

Male patients were significantly older and had higher height, weight, waist circumference, TGs, and LDL. Moreover, males had significantly lower BMI and HDL. There were no sex-related differences in terms of MetS prevalence or FBG (Table 4). When analyses were run separately for males and females, sex-related significant associations were found. For males, all MetS components were not statistically significant for worse WOMAC scores (Table 5). For females, there was a statistically significant association between the presence of increased high FBG with worse mean total and function WOMAC scores by 2.1 and 1.6 points, respectively (Table 6). Females also had a statistically significant association between having a reduced HDL and lower (i.e., better) mean total WOMAC scores by 3.3 points, WOMAC pain scores by 0.6 points, WOMAC stiffness scores by 0.3 points and WOMAC function scores by 2.4 points. The presence of high TG levels was also found to have lower mean WOMAC physical function score by 4.2 points in females only. The presence of high BP had no statistically significant association with worse WOMAC scores in female individuals.

**Table 1:** Participants’ baseline characteristics

	MetS not present (N = 5,070)	MetS (N = 930)	P value
**Age (years)**	37.5 ± 12.2	50.11 ± 12.2	< 0.001
**Sex (Female: Male)**	2,894:2,176	510:420	0.21
**Anthropometrics**			
Height (m) ± SD	1.64 ± 0.93	1.63 ± 0.1	0.003
Weight (Kg) ± SD	77.36 ± 17.22	91.3 ± 18.6	< 0.001
BMI (Kg/m^2^) ± SD	28.6 ± 5.9	34.3 ± 6.2	< 0.001
Waist circumference (cm) ± SD	86.15 ± 13.4	103.14 ± 11.9	< 0.001
**Metabolic Parameters**			
FBG (mmol/L) ± SD	4.9 ± 1.3	7.45 ± 3.2	< 0.001
Triglycerides (mmol/L) ± SD	1.1 ± 0.58	2.03 ± 0.99	< 0.001
LDL (mmol/L) ± SD	2.84 ± 0.86	2.98 ± 1.01	< 0.001
HDL (mmol/L) ± SD	1.48 ± 0.39	1.12 ± 0.29	< 0.001
**WOMAC Scores**			
Total, mean ± SD	21.05 ± 18.96	27.86 ± 22.7	< 0.001
Pain, mean ± SD	4.96 ± 4.4	6.4 ± 5.1	< 0.001
Stiffness, mean ± SD	2.03 ± 1.9	2.53 ± 2.16	< 0.001
Physical function, mean ± SD	14.04 ± 13.4	18.9 ± 16.2	< 0.001

SD: standard deviation; BMI: body mass index; FBG: fasting blood glucose; LDL: low-density lipoprotein; HDL: high-density lipoprotein; WOMAC: Western Ontario and McMaster Universities osteoarthritis index. Continuous variables were compared with the two-group t test, and categorical variables were compared with the chi square test

**Table 2:** Regression adjusted association of Metabolic Syndrome (MetS) with Western Ontario and McMaster Universities osteoarthritis (WOMAC) total and subscale scores

Outcome measure	Model 1*			Model 2**		
	MetS Coefficient (95% CI)	P value	R^2^	MetS Coefficient (95% CI)	P value	R^2^
Total WOMAC score	**3.34 (1.93, 4.75)**	< 0.001	0.08	1.23 (-0.21, 2.69)	0.094	0.1
WOMAC Pain	**0.69 (0.36, 1.01)**	< 0.001	0.07	0.2 (-0.13, 0.53)	0.232	0.09
WOMAC Stiffness	**0.21 (0.72, 0.36)**	< 0.001	0.05	0.04 (-0.11, 0.19)	0.59	0.07
WOMAC Physical Function	**2.44 (1.44, 3.44)**	< 0.001	0.09	1 (-0.03, 2.02)	0.058	0.1

*Model 1 is adjusted for age and sex. **Model 2 is adjusted for age, sex, and body mass index. CI: confidence interval; MetS: Metabolic Syndrome

**Table 3:** Association the individual components of the MetS ATP III definition with the Western Ontario and McMaster Universities osteoarthritis (WOMAC) index; adjusted for age, sex and body mass index

Outcome measures	High BP		High FBG		High Triglycerides		Low HDL		Model R^2^
	Coefficient (95% CI)	P value	Coefficient (95% CI)	P value	Coefficient (95% CI)	P value	Coefficient (95% CI)	P value	
**Total WOMAC**	1.2 (-0.2, 2.6)	0.09	**1.3 (0.47, 2.6)**	**0.04**	0.5 (-0.8, 1.7)	0.5	**-2.4 (-3.5, -1.3)**	**< 0.001**	0.1
**WOMAC Pain**	0.2 (-0.1, 0.5)	0.2	0.3 (-0.01, 0.6)	0.06	0.1 (-0.2, 0.4)	0.4	**-0.5 (-0.7, -0.2)**	**< 0.001**	0.09
**WOMAC Stiffness**	0.1 (-0.02, 2.6)	0.1	0.08 (-0.05, 0.2)	0.2	0.004 (-0.1, 0.1)	0.9	**-0.2 (-0.3, -0.1)**	**< 0.001**	0.07
**WOMAC Function**	0.5 (-1.3, 2.3)	0.6	**1 (0.05, 1.9)**	**0.04**	0.3 (-0.5, 1.2)	0.5	**-1.7 (-2.5, -0.9)**	**< 0.001**	0.1

**Table 4:** Participants’ baseline characteristics grouped by sex

	Males (N = 2,596)	Females (N = 3,402)	P value
**Age (years)**	39.8 ± 12.6	38.9 ± 13.3	0.013*
**Metabolic Syndrome (%)**	16.2%	15%	0.21
**Anthropometrics**			
Height (m) ± SD	1.72 ± 0.06	1.57 ± 0.05	< 0.001*
Weight (Kg) ± SD	85.3 ± 18	75.1 ± 17	< 0.001*
BMI (Kg/m^2^) ± SD	28.7 ± 5.6	30.1 ± 6.7	< 0.001*
**Metabolic Parameters**			
FBG (mmol/L) ± SD	5.38 ± 1.9	5.28 ± 1.9	0.06
Triglycerides (mmol/L) ± SD	1.38 ± 0.87	1.14 ± 0.6	< 0.001*
LDL (mmol/L) ± SD	3.01 ± 0.95	2.75 ± 0.82	< 0.001*
HDL (mmol/L) ± SD	1.26 ± 0.34	1.56 ± 0.39	< 0.001*
Waist circumference (cm) ± SD	93.5 ± 13.7	85.1 ± 14	< 0.001*
**WOMAC Scores**			
Total, mean ± SD	17.7 ± 16.5	25.4 ± 21.3	< 0.001
Pain, mean ± SD	4.2 ± 3.9	5.9 ± 4.8	< 0.001
Stiffness, mean ± SD	1.7 ± 1.7	2.4 ± 2.1	< 0.001
Physical function, mean ± SD	11.7 ± 11.6	17.2 ± 15.1	< 0.001

SD: standard deviation; BMI: body mass index; FBG: fasting blood glucose; LDL: low-density lipoprotein; HDL: high-density lipoprotein. Continuous variables were compared with the two-group t test, and categorical variables were compared with the chi square test

**Table 5:** Association of the male individual components of the MetS ATP III definition with the Western Ontario and McMaster Universities osteoarthritis (WOMAC) index; adjusted for age and body mass index

Outcome measures	High BP		High FBG		High Triglycerides		Low HDL		Model R^2^
	Coefficient (95% CI)	P value	Coefficient (95% CI)	P value	Coefficient (95% CI)	P value	Coefficient (95% CI)	P value	
**Total WOMAC**	0.9 (-0.9, 2.7)	0.31	1.2 (-0.5, 2.9)	0.16	1.2 (-0.2, 2.7)	0.09	-1 (-2.5, 0.33)	0.1	0.013
**WOMAC Pain**	0.2 (-0.23, 0.6)	0.36	0.36 (-0.4, 0.8)	0.09	0.3 (-0.04, 0.7)	0.09	-0.3 (-0.6, 0.05)	0.09	0.014
**WOMAC Stiffness**	0.18 (-0.002, 0.4)	0.053	0.14 (-0.03, 0.3)	0.11	0.03 (-0.12, 0.2)	0.7	-0.06 (-0.2, 0.08)	0.37	0.006
**WOMAC Function**	0.6 (-0.7, 1.8)	0.4	0.7 (-0.5, 1.9)	0.24	0.9 (-0.9, 2)	0.8	-0.7 (-1.7, 0.25)	0.15	0.012

CI: confidence intervals; BP: blood pressure; FBG: fasting blood glucose; HDL: high-density lipoprotein, WC: waist circumference

**Table 6:** Association of the female individual components of the MetS ATP III definition with the Western Ontario and McMaster Universities osteoarthritis (WOMAC) index; adjusted for age and body mass index

Outcome measures	High BP		High FBG		High Triglycerides		Low HDL		Model R^2^
	Coefficient (95% CI)	P value	Coefficient (95% CI)	P value	Coefficient (95% CI)	P value	Coefficient (95% CI)	P value	
**Total WOMAC**	1.4 (-0.6, 3.5)	0.17	**2.1 (0.2, 3.9)**	**0.032**	-0.2 (-2.2, 1.7)	0.8	**-3.3 (-5, -1.6)**	**< 0.001**	0.11
**WOMAC Pain**	0.2 (-0.27, 0.7)	0.4	0.4 (-0.5, 0.8)	0.08	-0.05 (-0.5, 0.38)	0.8	**-0.6 (-1, -0.24)**	**0.001**	0.1
**WOMAC Stiffness**	0.05 (-0.15, 0.3)	0.62	0.08 (-0.1, 0.27)	0.41	-0.003 (-0.2, 0.2)	0.9	**-0.3 (-0.5, -0.14)**	**< 0.001**	0.08
**WOMAC Function**	1.2 (-0.3, 2.7)	0.11	**1.6 (0.27, 2.9)**	**0.018**	**-4.2 (-6.5, -1.9)**	0.8	**-2.4 (-3.5, -1.2)**	**< 0.001**	0.12

CI: confidence intervals; BP: blood pressure; FBG: fasting blood glucose; HDL: high-density lipoprotein, WC: waist circumference

## Discussion

We conducted a population-based study in Qatar to report on the epidemiologic association between MetS and knee symptoms. Applying the ATP III criteria, we found that 15.5% of the 6,000 participants in our study had MetS. Moreover, the analysis of patient demographics revealed age- and sex-related difference in patient demographics which prompted us to conduct multivariable regression analysis adjusted for these factors. The association between MetS and WOMAC total and subscale scores was significant in the analyses without BMI adjustment, but when adjusted for BMI, the association was no longer significant. However, when examining the association of individual MetS criteria components with WOMAC scores, there were inconsistent sex-based associations even after adjusting for BMI. These findings have important epidemiologic implications.

In our population-based study, we found that 15.5% of participants fulfilled the criteria for having MetS. The rate of MetS in this large population-based study is considerably lower compared to the 26.5% rate reported in a previous survey of the Qatari population through primary health centers [22]. Although both surveys utilized the ATP III criteria to define MetS, the lower rate in our study can be attributed to multiple reasons. First, we utilized population-based data, which included both healthy and unhealthy individuals. Data from the primary care centers would likely include more individuals who were obese and with health problems seeking medical care. Second, since 2013, the Ministry of Health in Qatar has implemented multiple initiatives to address the obesity epidemic in the country and it is possible that the rate of MetS has declined as a result of these initiatives. For instance, obesity prevention has been one of main objectives of Qatar’s national health strategy for the years 2018 through 2022 [29]. Another notable initiative is the establishment of the National Obesity Treatment Center in 2017 which provides multidisciplinary care for obese individuals [30].

We found that MetS was significantly associated with worse knee symptoms when compared to participants without MetS. However, this association was not sustained after controlling for BMI. Several population-based studies support our findings. In a longitudinal study of 985 participants, Pan et al. reported that having MetS was significantly associated with worse WOMAC pain scores [16]. However, the association was not significant once BMI was accounted for. Likewise, in a cross-sectional study of 952 women, Sanchez-Santos et al. found that painful radiographic KOA was not significantly associated with MetS after adjusting for BMI [15]. In the Framingham study on 991 subjects with KOA, similar findings were reported by Niu et al. [17]. However, the role of MetS on worsening knee symptoms remains controversial with other studies supporting the significant role of MetS in knee joint degeneration. Shin et al. found in a South Korean national survey on 2,363 subjects that knee pain scores were significantly associated with increasing accumulation of MetS components while adjusting for BMI or weight [14]. In an epidemiologic study on 5,764 subjects from a Chinese population, there was a positive association between MetS with increasing prevalence of KOA despite adjusting for age, sex, and BMI [12]. In study on 60 Egyptian patients with KOA, Afifi et al. reported a significant increase in WOMAC pain scores severity when MetS was present despite adjusting for BMI [13]. This heterogeneity in the findings of these studies suggest that the association may be more complex and deserves further investigation.

Regarding the effect of the metabolic components of MetS, our study displayed inconsistent results. In this cross-sectional study, high FBG levels predicted significantly worse WOMAC function scores only in female individuals. An unexpected finding was that low HDL and high TG levels were associated with better WOMAC scores in females. For male individuals, there were no correlations between the metabolic components and knee symptoms. Nevertheless, these statistically significant findings were not beyond minimum clinically important differences for WOMAC scores [31]. The association between MetS components and worse knee symptoms remains unclear in the current literature with conflicting results. Multiple cohort studies have found that MetS components did not predict worse knee outcomes. In the multivariate regression analysis of the Framingham Osteoarthritis study, Niu et al. [17] found that none of the MetS components were associated with increased KOA incidence. Similarly, a cohort study of 6,274 individuals from Finland did not find any association between MetS components and the incidence of KOA [32]. In contrast, Askari et al. [33] reported in an Iranian registry study that the metabolic components of MetS were significantly associated with increased risk of developing OA. Recent meta-analyses on MetS and OA reported conflicting results for [34] and against [35] the contribution of MetS components to worse knee outcomes. As such, further well-designed prospective studies are needed to elucidate the role of MetS components in the development of knee pain and KOA.

While our study is the first in Qatar and the largest in the Middle East to examine this relationship and provide insight, there are several notable limitations that should be acknowledged. First, this is a cross-sectional study and indicates association not causation of increased knee scores as a result of MetS. Second, we examined the association between MetS and knee symptoms. While these symptoms may be the hallmark of knee osteoarthritis, we did not have radiographs on participants to determine if radiographic knee osteoarthritis was present. Third, the study occurred in Qatar and may not represent all countries in the middle east, which may have different obesity profiles. Finally, while the Qatar Biobank processes of data collection are very rigorous, it is possible that there would be errors in the data collection.

In conclusion, this population-based study in a Middle Eastern country demonstrated that after adjustment for BMI, neither MetS nor its individual parameters were associated with clinically relevant worse knee symptoms. Further clinical and radiographic assessment of the participants of this study would be paramount to document the impact of obesity and MetS on KOA.

## Data Availability

The datasets generated and/or analyzed during the current study are not publicly available as per the IRB approval but are available from the corresponding author on reasonable request.
